# Unpacking Investor Psychology: A Systematic Review and Meta-Analysis of Behavioural Biases Shaping Investment Decisions*

**DOI:** 10.12688/f1000research.168166.1

**Published:** 2025-10-21

**Authors:** Herath Mudiyanselage Methma Ashvini Herathmenike, Narayanage Jayantha Dewasiri, Amila Munasinghe

**Affiliations:** 1 University of Kelaniya Faculty of Commerce and Management Studies, Kelaniya, Western Province, Sri Lanka; 2 Department of Accountancy and Finance, Faculty of Management Studies, Sabaragamuwa University of Sri Lanka, Belihuloya, Sabaragamuwa Province, Sri Lanka; 3 Adjunct Professor, Qasim Ibrahim School of Business, Villa College, Malé, Male, Maldives; 4 Department of Accountancy, Faculty of Commerce & Management Studies, University of Kelaniya, Kelaniya, Western Province, Sri Lanka

**Keywords:** Behavioural Biases, Investor Psychology, Investment Decisions, Systematic Review, Informal Markets

## Abstract

**Background:**

This study systematically examines how behavioural biases affect investment decisions across formal and informal financial markets, with a focus on emerging economies. It aims to map the evolution of the field, highlight neglected cognitive biases, and uncover geographical and methodological gaps in current research.

**Methods:**

A rigorous Systematic Literature Review (SLR) was conducted using the PRISMA protocol, analysing 63 empirical studies from the Scopus database. Advanced bibliometric tools, VOSviewer and Biblioshiny, were employed to identify key trends, thematic clusters, and shifts in research focus in behavioural finance.

**Results:**

The review reveals a heavy concentration of studies on biases such as overconfidence, herding, and loss aversion, primarily within formal market settings and South Asian contexts. Many other essential biases, including regret aversion, anchoring, and emotional influences, are still rarely studied. Very few studies test real-world solutions, such as education, digital tools, or reminders, that may help people make better financial decisions. Visual and longitudinal mapping demonstrate a rising academic interest after 2016, but expose a lack of representation from many regions and emerging behavioural dynamics. Research on informal investing and diverse cultural settings is also limited.

**Conclusion:**

This review highlights that many investors, especially in emerging economies, are guided by emotions and mental shortcuts rather than careful reasoning. While interest in this topic has grown, most research still focuses on a few well-known biases and limited regions. Many significant biases, such as regret aversion and anchoring, remain underexplored. There is also very little evidence on what helps reduce these biases in real-world settings. This study advocates for more diverse, practical, and inclusive research that can help investors make informed financial decisions, particularly in informal markets where support is limited.

## Introduction

Making smart investment decisions can be challenging. It is not just about looking at financial numbers. Traditional finance theories like the Efficient Market Hypothesis by. ^1^ It suggests that investors always act rationally based on the available information. However, behavioural finance takes a different perspective. It demonstrates how emotions, mental shortcuts, and biases frequently influence people’s investment choices. ^2^ These psychological factors can lead investors to make decisions that are not always logical, potentially resulting in poor financial outcomes.

This influence is even more substantial in financial schemes that are not regulated by official authorities. In such setups, investors often rely on advice from friends, social pressure or simple mental rules (heuristics) to decide where to invest. ^3^ Since these schemes lack proper tools for assessing risk, common biases like loss aversion, herding behaviour and mental accounting become more powerful. ^4^ For example, loss aversion means people care more about avoiding losses than gaining profits. As a result, they might keep losing investments longer than they should. ^5^

Herding behaviour is another common bias. It means that people tend to follow what others are doing instead of thinking for themselves. ^6^ This usually occurs due to the fear of missing out (FOMO) or a desire to fit in socially. It can lead to investment bubbles and significant financial losses. ^7^ Additionally, how an investment is presented, known as framing, can strongly affect how risky it appears. The same offer can look either safe or dangerous, depending on how it is described. ^8^

Understanding how people see risk is especially crucial in financial settings because people rely on limited or secondhand information like stories, personal experiences or word of mouth to judge risk. ^9^ This can cause them to either underestimate or overestimate the danger involved, leading to poor decisions. ^10^ This study expands the discussion of behavioural finance to include developing countries where investments are common but risky. ^11^ This review aims to synthesise existing academic work, identify key behavioural patterns, and highlight gaps in the literature that warrant further investigation. By doing so, this study contributes to a more comprehensive understanding of how cognitive and psychological factors shape investor behaviour, particularly in specific settings.

To achieve this goal, the study is guided by the following research questions, aiming to provide a clear understanding of the theoretical background and to explore existing empirical research in the areas of behavioural finance and investment decision-making.

1. Which core psychological theories and behavioural biases best explain how investors decide where to put their money?
2. How do different behavioural biases shape investor choices across formal and informal markets and diverse cultural settings?
3. How have topics, methods and geographical coverage in behavioural bias research on investing changed from 1999 to 2025?
4. What unstudied biases, regions or intervention strategies remain, and how can future work close these gaps and help investors make better decisions?

### What makes this study different from past reviews on behavioural biases

A comparison between this study and earlier systematic reviews on investment biases is presented in Table I.

**Table I.** This study vs. earlier systematic reviews on investment biases.

Study	Difference from current review
^26^	This study looks only at formal markets in developed countries and focuses on a few common biases like overconfidence. It does not include informal settings or show how research has changed over time. It uses a basic method without visual tools. Our review includes both formal and informal markets, more types of biases, trends from 1999–2025 and visual tools for deeper analysis.
^27^	This review is limited to formal markets and mainly covers popular biases. It does not look at different cultures or how the research has changed over time. Their method is simple. Our review covers more markets, compares cultures, explores trends over 26 years and uses tools like VOSviewer and PRISMA to find deeper patterns.
^28^	This study does not explore trends of biases and it does not use tools to analyze. Our review includes a wider range of biases, informal investing and research patterns over many years and countries.
^29^	This study looks only at emerging markets and includes fewer papers. It does not explore how research has changed over time. It also uses a basic review method. Our study looks at both formal and informal markets, covers 26 years of research and uses visual tools to show patterns.
^30^	This review does not separate different markets, does not study changes over time and uses only simple methods. Our review is more detailed, uses strong tools and shows how the field developed.

Source: Authors developed.

## Methods

This article follows a Systematic Literature Review (SLR) approach, which requires setting clear guidelines before defining which studies to include or exclude and how to analyse them. ^12^ To achieve this, we created a detailed plan before commencing our literature search. In medical research, SLRs are often reported using a standard called PRISMA (Preferred Reporting Items for Systematic Reviews and Meta-Analyses), which is widely accepted and recommended. ^13^ However, in social sciences, such a structured reporting method is lacking. As a result, many researchers rely on less formal or subjective approaches. ^12, 14^ In contrast, our study employed a more rigorous approach, utilising the PRISMA article selection flow chart to ensure that the article selection process was both objective and systematic.

### Search strategy

The article selection process in this study adhered to the four steps outlined in the PRISMA flow diagram: identification, screening, eligibility and inclusion. These steps were followed systematically during the selection of articles. During the identification phase, we relied exclusively on the Scopus database to identify and select relevant research articles. Scopus was chosen because it offers one of the most comprehensive and reliable collections of peer-reviewed literature, particularly in areas related to behavioural finance, psychology, and investment decision-making. It provides consistent indexing, rich bibliometric data and advanced filtering tools, which were crucial for applying our inclusion criteria and conducting visual analyses using tools like VOSviewer and Biblioshiny.

We did not include Web of Science or Google Scholar for several reasons. Web of Science, while reputable, has more limited coverage in social sciences and regional studies, particularly from developing countries, which are central to our review. Google Scholar, on the other hand, includes a wide variety of sources but lacks transparency in its indexing process and often returns non-peer-reviewed content, such as blog posts, teaching materials, or duplicate records. This can reduce the reliability and consistency needed for a systematic review. By focusing solely on Scopus, we ensured that our review utilised high-quality, peer-reviewed, and methodologically traceable sources, making it more focused, rigorous, and credible.

The concepts employed to search were “Behavioural Biases” and “Investment Decisions”, which were strategically chosen to capture relevant studies in the field.

The main research question addressed in this systematic review is “What behavioural biases influence investment decisions and how have these biases been conceptualised and measured in existing literature?” The search was conducted within the Scopus database, focusing on article titles, abstracts and keywords. The search was performed in May 2025. A detailed overview of the search strategy and the combination of keywords used in this study is provided in Table II.

**Table II.** Search strategy.

Search strategy	#1 AND #2
Concepts	Keywords
#1 Behavioural Biases	“Behavioural Bias” OR “Behavioural Biases” OR “Behavioral Bias” OR “Behavioral Biases” OR “Investor Psychology”
#2 Investment Decisions	“Investment Decisions”

Source: Authors constructed.

The search process for this study was designed using a straightforward method. Keywords were combined using the word “AND” to link different concepts, while similar words were connected using “OR” to broaden the search. This method enabled us to locate 269 articles from the Scopus database. All article details, including titles, abstracts, keywords, author names, affiliations, journal names, number of citations, and publication years, were exported into a Microsoft Excel sheet. After this, we checked for duplicate articles and removed them.

The next step was screening. At this stage, we excluded articles if their titles or abstracts did not match our criteria. As suggested by. ^15^ This step is essential for selecting only the most relevant studies.

The inclusion criteria for this review were:

• The article must be an empirical study.
• It must be published in an academic journal.
• It must be written in English.
• It must focus on Behavioural Biases and Investment Decisions.
• It must be published between 1999 and 2025.

We chose 1999 as the starting year because the earliest article found in Scopus using our keywords (“Behavioural Bias”, “Behavioural Biases”, “Behavioural Bias”, “Behavioural Biases”, “Investor Psychology” and “Investment Decisions”) was from that year. We extended the period to 2025 to include the most recent research available.

Each author reviewed the titles and abstracts independently. If there were any disagreements about excluding an article, they were resolved through discussion and mutual agreement. In total, we excluded 93 articles that were not in the English language, articles in press, book chapters, reviews, conference papers, books, retracted articles, errata, notes, and articles from Trade Journals. Following this, the remaining 176 articles were selected for full-text review, the next stage in the PRISMA flow diagram.

At this stage, the articles had already been screened, so it was more appropriate to assess how well they reported their research methods to decide if they should be included. ^15^ This step was justified because one of our inclusion criteria was that the study must be an “empirical study.” Therefore, we examined elements such as the study population, methodology, research design and context. These aspects helped us identify reasons to exclude some articles, such as unclear methods or missing information. ^15^

Each article was evaluated independently based on these criteria. We found that some studies were based on opinions or qualitative reviews, had unclear methods or lacked enough detail about how the research was conducted. These articles were excluded after group discussion and agreement.

**Figure 1.**
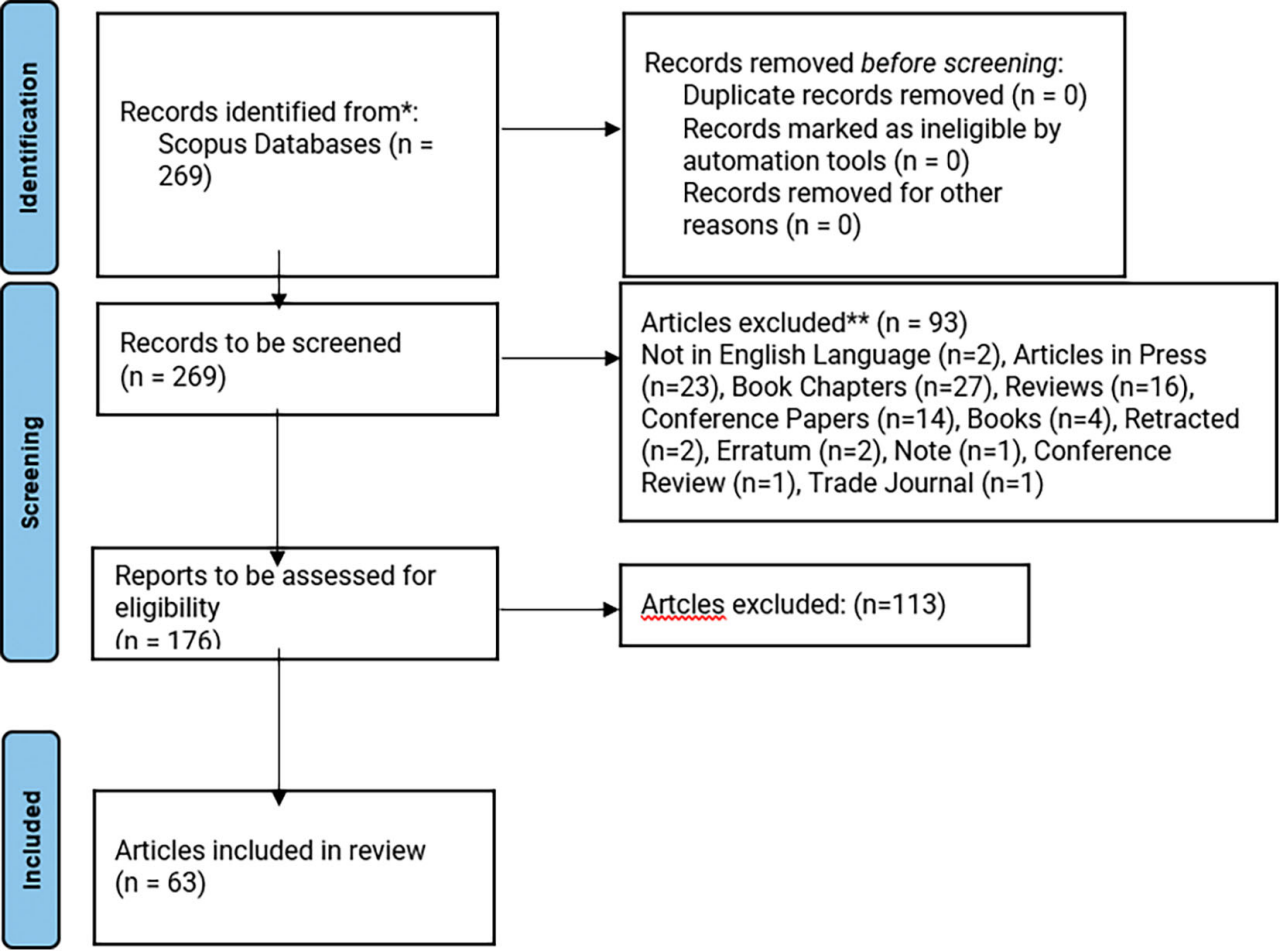
PRISMA article selection flow diagram. Source: The Authors constructed.

In total, 113 articles were excluded at this stage, and 63 articles were selected for the final review. These selected articles are listed in Table II, and the article selection process is shown in Figure 1. We then updated the data in a Microsoft Excel sheet and used VOSviewer and RStudio software to create maps showing keyword and term co-occurrences, as well as other analyses. These visual maps helped us identify the main themes across the selected studies. The keyword co-occurrence map highlighted key topics based on the keywords provided by the authors. In contrast, the term co-occurrence analysis provided further insights by examining the critical terms in the titles and abstracts.

## Results

**Figure 2.**
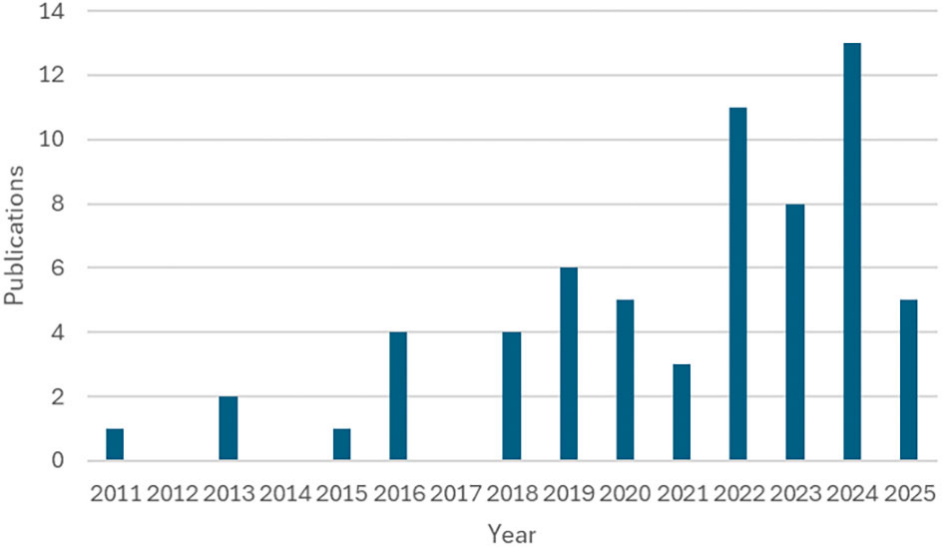
Year-wise research article distribution. Source: Authors developed.

Figure 2 shows the number of publications each year from 2011 to 2025. Although 1999 was selected as the starting year for this review, no publications were found between 1999 and 2010. The first publication appeared in 2011, and the number remained low until 2015. From 2016 onward, the number of publications began to increase gradually. A noticeable rise is observed from 2019, with the highest number of publications in 2024. There is a slight decrease in 2025. Overall, Figure 2 illustrates a growing research interest in the topic over time, particularly in recent years. However, the limited number of studies before 2016 suggests a clear research gap. The lack of attention in earlier years indicates that some aspects of the topic may still be underexplored, offering opportunities for further investigation and contributions to the field.

Table III shows the number of publications by country in this study. India stands out with the highest number of publications (n = 93), followed by Pakistan (n = 33). China ranks third with 10 publications. Countries such as Indonesia and Nepal (each with 8), Malaysia and Morocco (each with 6), and Turkey (with 5) have made moderate contributions. Additionally, countries such as Bangladesh, Iraq, Jordan, Poland, Tunisia, the United Kingdom, and the United States each contributed three publications. A few countries, such as Libya, Oman, and Saudi Arabia, had two publications. In contrast, others, including Finland, Guinea, Hungary, Latvia, Papua New Guinea, South Africa, and Thailand, contributed only one each.

**Table III.** Country-wise scientific production.

Country	Frequency
INDIA	93
PAKISTAN	33
CHINA	10
INDONESIA	8
NEPAL	8
MALAYSIA	6
MOROCCO	6
TURKEY	5
BANGLADESH	4
IRAQ	3
JORDAN	3
POLAND	3
TUNISIA	3
UK	3
USA	3
LIBYA	2
OMAN	2
SAUDI ARABIA	2
FINLAND	1
GUINEA	1
HUNGARY	1
LATVIA	1
PAPUA NEW GUINEA	1
SOUTH AFRICA	1
THAILAND	1

Source: Authors through Biblioshiny.

The data suggest that a significant share of publications originates from South Asian countries, especially India and Pakistan. For example, studies from India have addressed topics such as behavioural biases in investment decisions ^16^ and individual investor psychology. ^17^ Similarly, work from Pakistan has explored decision-making influences among individual investors. ^18^ These trends indicate a growing academic interest in behavioural finance within the region, while contributions from other parts of the world remain limited.

However, this also highlights a clear research gap. Much of the existing literature is concentrated in a small number of countries, leaving many regions underrepresented or unrepresented in academic discussions. As a result, we lack insights into how cultural, economic and social differences may influence investor behaviour globally. To build a more complete and inclusive understanding of behavioural finance, future research should focus on involving countries and regions that currently have low or no publication activity in this field.

Table IV presents the total citations (TC) and average article citations for each country included in the study. India leads in total citations with 463, averaging 25.7 citations per article. This high output is not only due to the large number of publications but also to the visible impact of the work. Pakistan ranks second with 110 total citations and an average of 18.3 citations per article.

**Table IV.** Most cited countries.

Country	TC	Average article citations
INDIA	463	25.70
PAKISTAN	110	18.30
NORWAY	53	53.00
INDONESIA	49	24.50
MALAYSIA	45	22.50
CHINA	30	7.50
USA	19	19.00
MALTA	17	17.00
TURKEY	16	16.00
BANGLADESH	14	14.00
UNITED KINGDOM	8	8.00
JORDAN	5	5.00
MOROCCO	2	1.00
OMAN	1	1.00
POLAND	1	1.00

Source: Authors through Biblioshiny.

Interestingly, Norway, despite having fewer total publications, displayed the highest average citation rate at 53.0, suggesting highly impactful work. Other countries, such as Indonesia (24.5), Malaysia (22.5), and the USA (19.0), also showed strong average citation performance. Countries such as Malta, Turkey, and Bangladesh followed closely, with moderate citation averages. In contrast, countries such as Morocco, Oman, and Poland had the lowest citation averages at 1.0, possibly due to fewer or more recent publications.

This distribution again demonstrates that South Asia, specifically India and Pakistan, not only contributes a high volume of publications but also achieves a notable citation impact. However, some countries with fewer publications achieve higher average citation rates, suggesting that a small number of their studies have made a substantial impact in the field.

This highlights a meaningful research gap. While countries like India and Pakistan dominate in both quantity and impact, many other nations are either underrepresented or have low citation averages. This could mean that essential insights from those regions are being overlooked or that their research is not being disseminated to a broader audience. Moreover, the success of countries like Norway, with high citation averages, shows that impactful research does not always require a high number of publications. Therefore, there is a need for more research from underrepresented countries, as well as support to make their work more visible and accessible in the global academic community.

*Literature classification*

**Figure 3.**
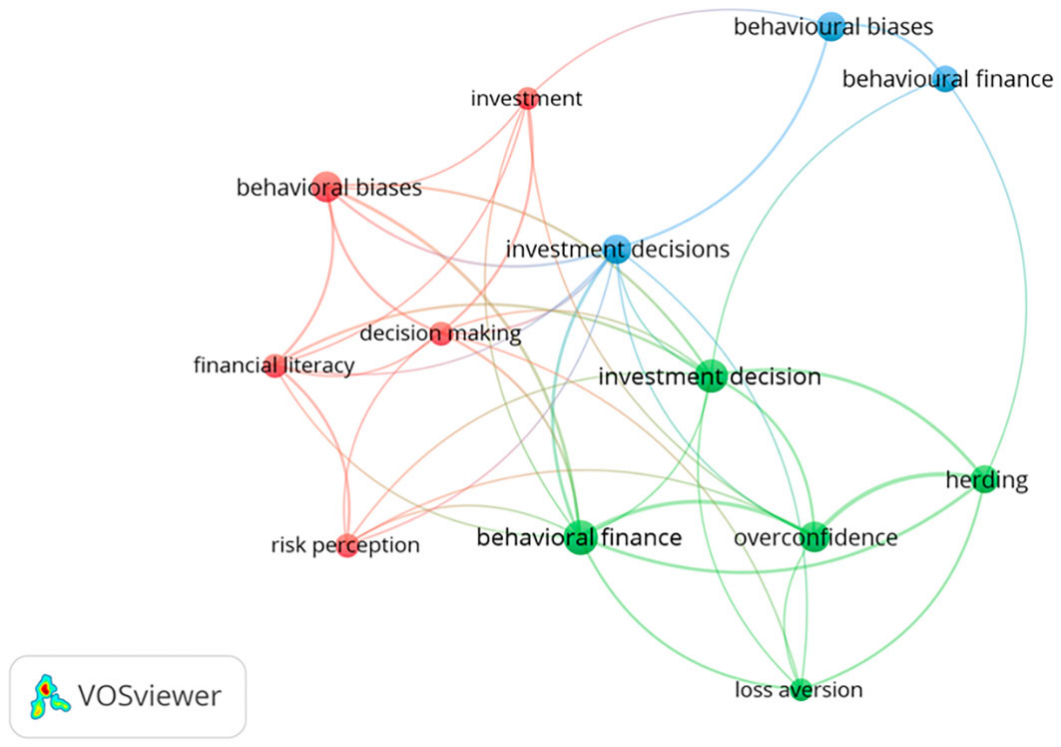
Keywords co-occurrence network visualisation map. Source: Authors generated through VOSviewer.

Organising the results is an important step in understanding the actual work that has been done to meet the research objectives. ^19, 20^ Since the primary aim of this study was to review existing academic research, identify common behavioural patterns, and uncover gaps that require further exploration, this section presents a classification of the findings based on these goals. To support this, we employed a keyword co-occurrence network analysis using VOSviewer software, a valuable method for identifying key focus areas within a field of study. The results of this analysis are presented in Figure 3.

The size of each node in the keyword co-occurrence visualisation map represents the frequency at which a keyword appears. A larger node indicates a higher number of occurrences. Based on our analysis, the keywords ‘behavioural finance’, ‘investment decision’, ‘overconfidence’, and ‘behavioural biases’ appeared as larger nodes in the map ( Figure 3), indicating that they are frequently mentioned in the reviewed studies. The frequent appearance of behavioural biases and overconfidence is linked to the widespread discussion around behavioural finance, which helps explain its prominence. Similarly, investment decisions often appear in the literature because research has shown that behavioural biases play a significant role in decision-making.

Figure 3 also presents three main keyword clusters, each shown in a different colour, with each cluster containing a set of related keywords. Table V provides the number of terms in each cluster, indicating that research on behavioural biases in investment decisions spans various focus areas. Keywords grouped within the same cluster are likely to represent closely related topics. Clusters one and two, which include the highest number of keywords (as shown in Table V), suggest that these areas are central to research on behavioural biases in investment decisions. The key themes highlighted in these clusters include behavioural biases, decision-making, financial literacy, investment, risk perception, behavioural finance, herding, investment decisions, loss aversion, and overconfidence.

**Table V.** Clusters of keywords.

Cluster	No. of items	Common theme (AI assisted)	Item
Red Cluster	5	Behavioral foundations of financial decision-making	behavioral biases, decision making, financial literacy, investment, risk perception
Green Cluster	5	Psychological drivers of investment choices	behavioral finance, herding, investment decision, loss aversion, overconfidence
Blue Cluster	3	Core ideas in behavioral finance	behavioural biases, behavioural finance, investment decisions

Source: Authors generated.

Some important behavioural biases, such as anchoring, mental accounting, regret aversion, and a range of emotions beyond overconfidence and herding, are either missing or appear only at the edges of past studies. This indicates that they have not been thoroughly explored yet. Future studies can expand the variety of biases being studied and examine more closely how these lesser-known mental shortcuts and emotional reactions influence the way people make investment decisions.

**Figure 4.**
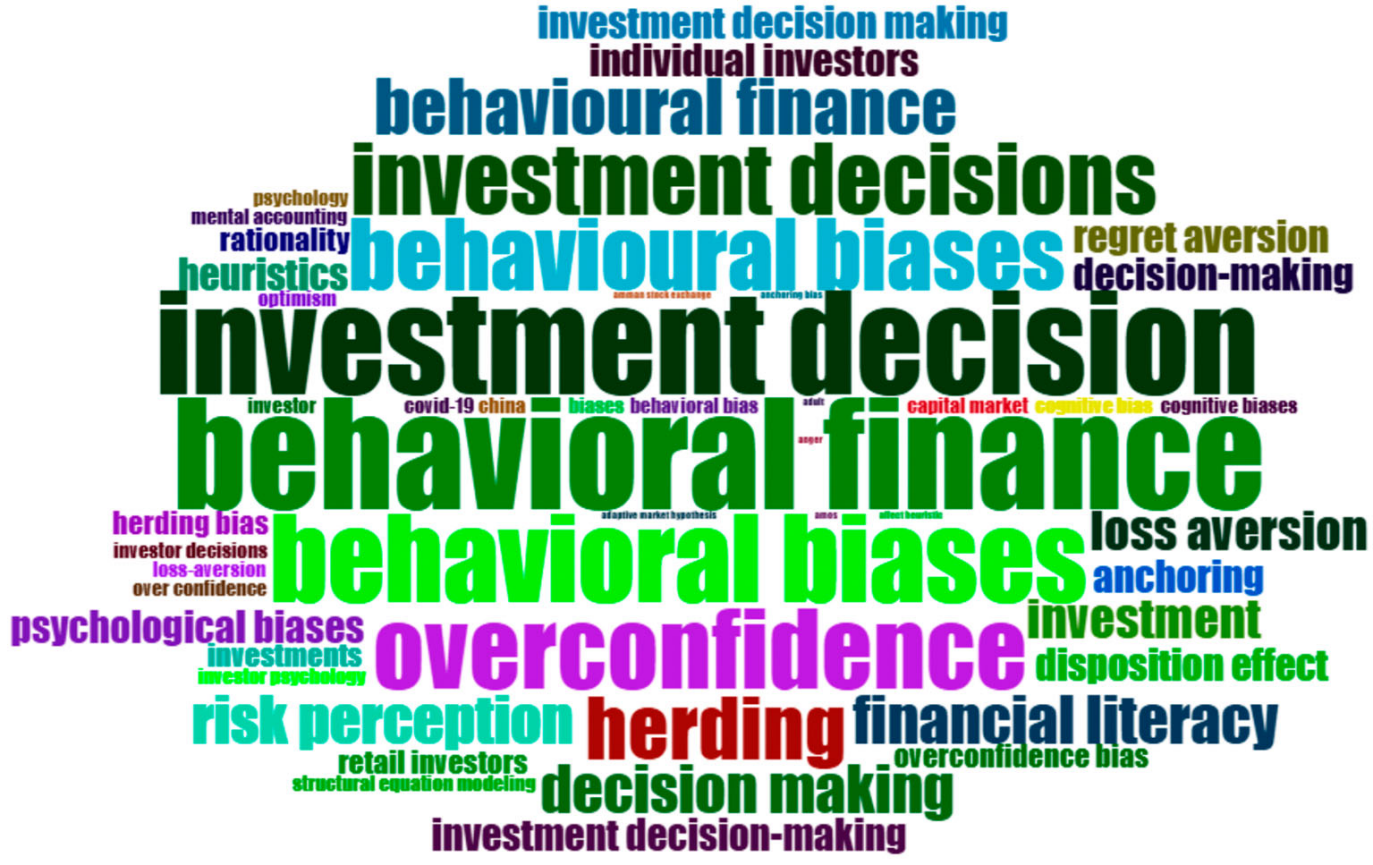
Word cloud. Source: Authors through Biblioshiny.

The word cloud and tree map show the most common and important keywords used in the research articles studied. These visuals helped us quickly understand what topics researchers focus on the most. In the word cloud, larger words indicate that these keywords appear more frequently in the studies. The biggest words like “investment decision”, “behavioural finance”, “behavioural biases”, “overconfidence” and “herding” show in Figure 4 that these are the main areas of interest for researchers. Other common topics include “financial literacy”, “risk perception”, “decision making” and “loss aversion”. These are all connected to how people make financial decisions and the factors that influence their behaviour.

**Figure 5.**
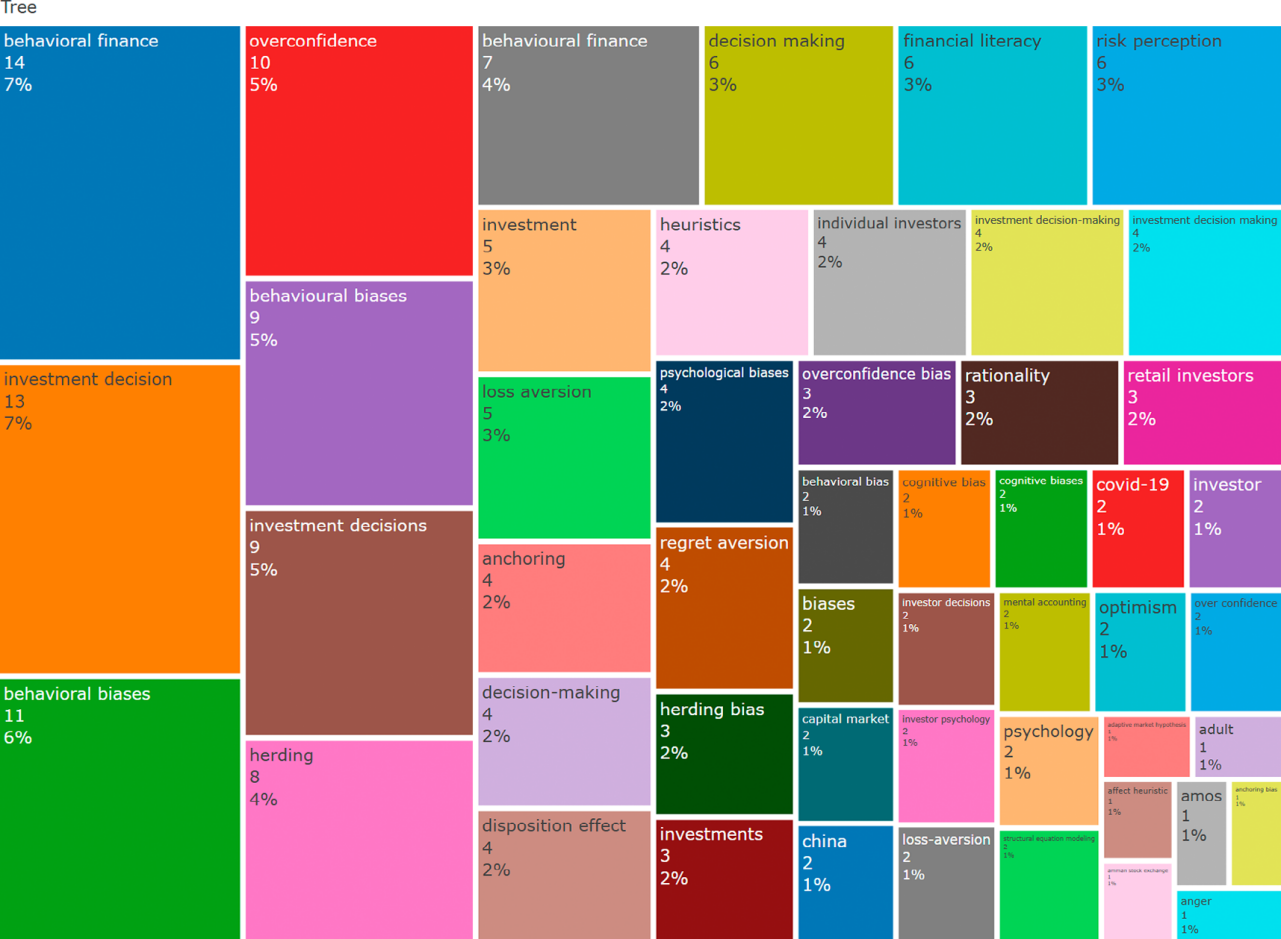
Tree map. Source: Authors through Biblioshiny.

The tree map in Figure 5 displays the exact keywords in a box format. Each box’s size indicates how often the word appears, and the colours make it easy to separate the topics. Again, we observed that “behavioural finance” and “investment decision” are the largest, confirming that these are the most talked about topics. We also observed a wide variety of related ideas like “psychological biases”, “anchoring”, “regret aversion”, “mental accounting” and even “Covid-19”, which shows that some studies have looked at recent global events too.

These visuals indicate that the field of behavioural finance places a strong emphasis on understanding how people think, feel, and act when making financial investment decisions. They also reveal that biases and emotions, such as overconfidence or regret, can significantly influence investment choices.

Furthermore, the term co-occurrence network visualisation map created using VOSviewer ( Figure 8) offers a more detailed analysis compared to the keyword co-occurrence map. While keyword analysis focuses only on the assigned keywords, the term co-occurrence map examines the content within the titles and abstracts of the studies. This deeper level of analysis helps uncover the specific areas that have been most frequently explored in research related to behavioural biases in investment decisions.

**Figure 6.**
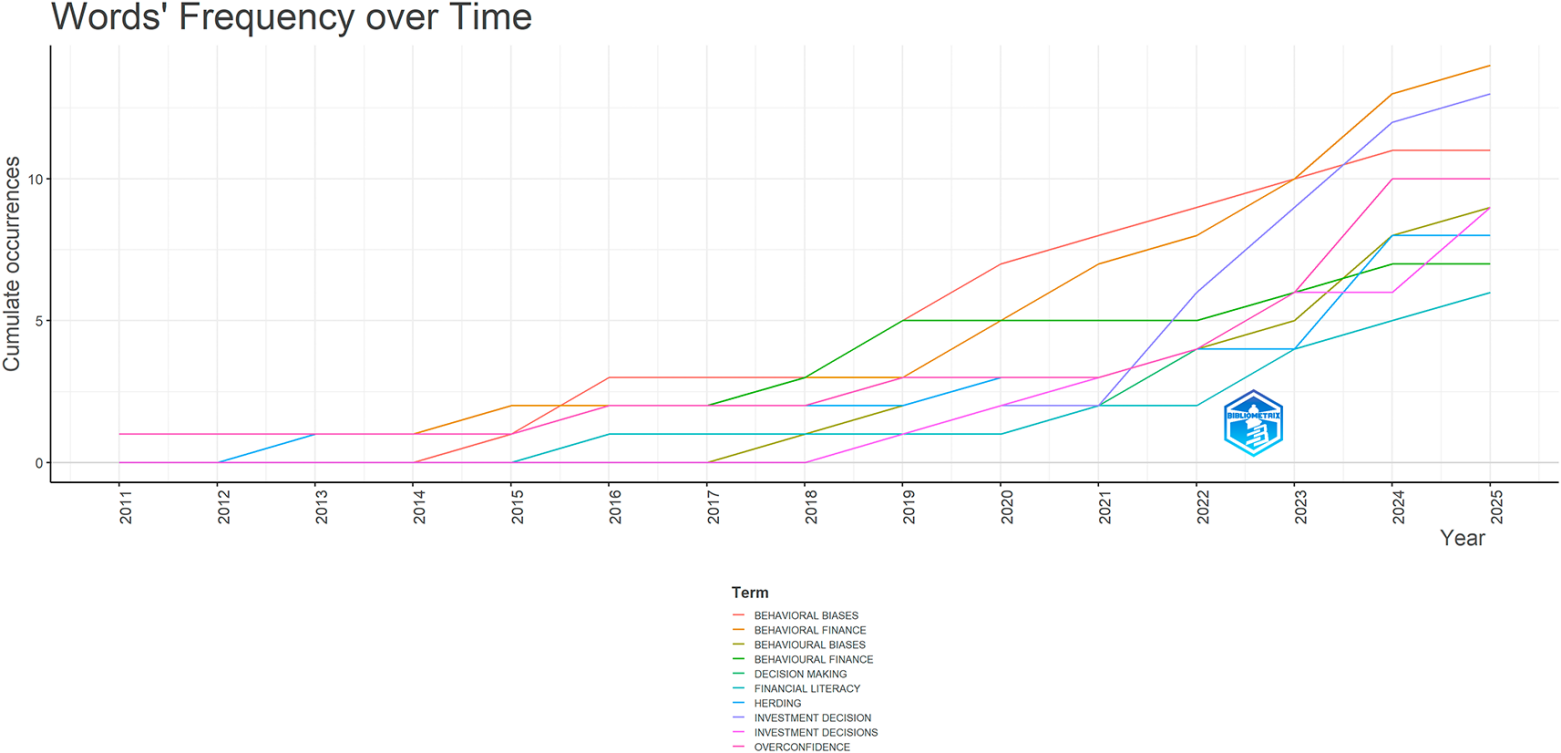
Word’s frequency over time. Source: Authors through Biblioshiny.

**Figure 7.**
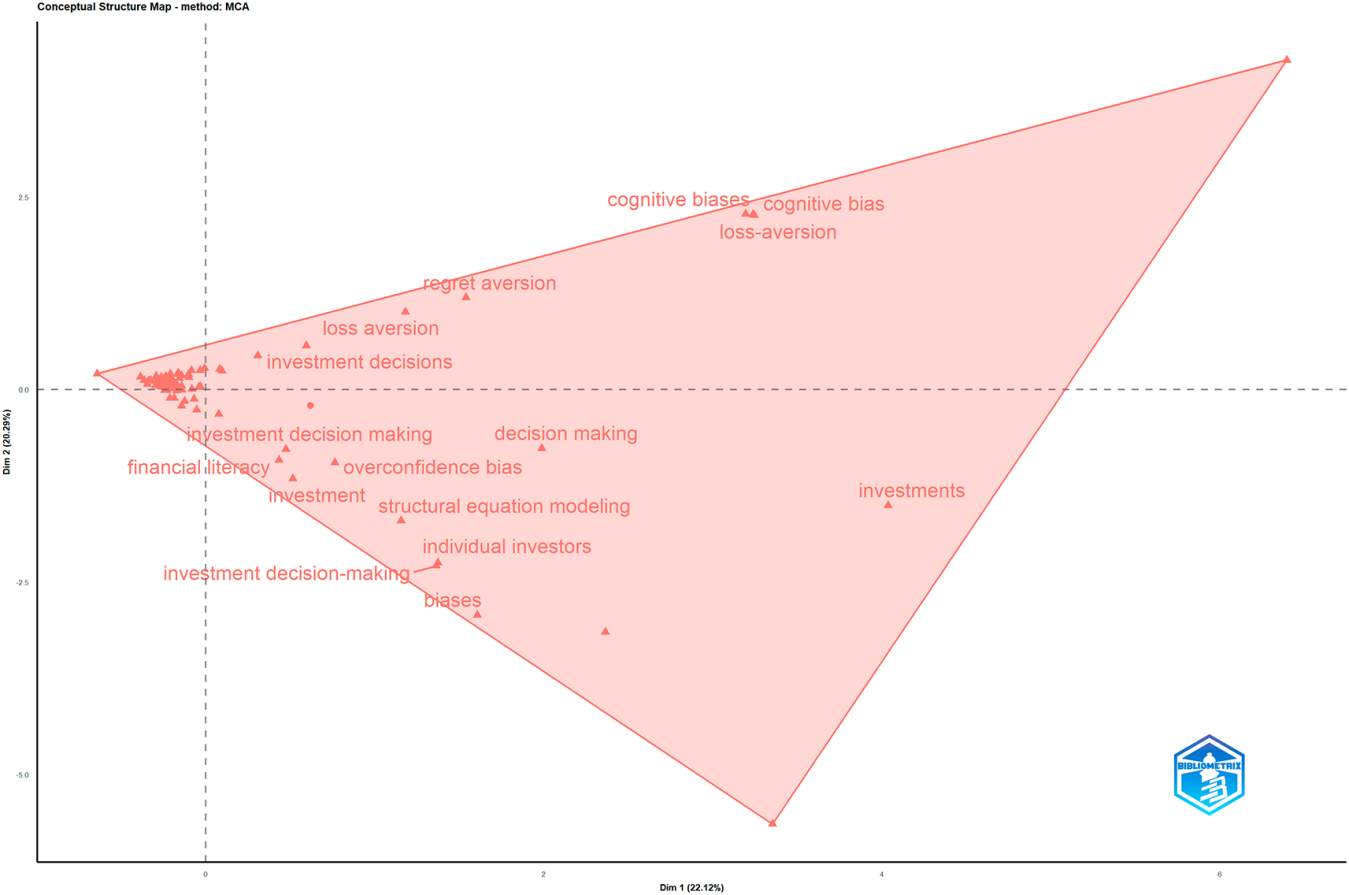
Conceptual structure map. Source: Authors through Biblioshiny.

**Figure 8.**
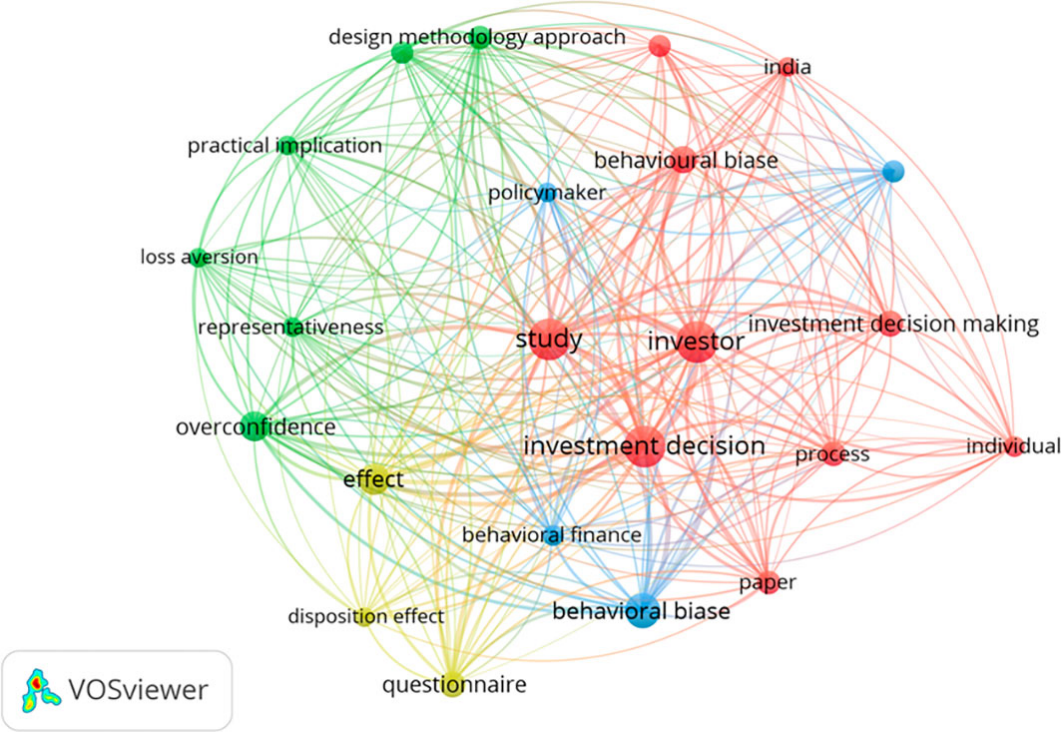
Term co-occurrence network visualization map. Source: Authors generated through VOSviewer.

This highlights a clear research gap, as the field heavily concentrates on a small group of well-known concepts, such as overconfidence and herding. While these are undoubtedly significant, many other biases, such as anchoring, mental accounting, regret aversion, and emotional factors, are less frequently observed or mentioned only briefly. These underexplored areas deserve more attention. Future research could make a meaningful contribution by broadening the scope of behavioural biases studied and by investigating how emerging challenges and emotional factors influence financial decision-making today.

Figure 6 shows how often some crucial words have been used in research papers from 2011 to 2025. Each line represents a keyword, and the way the lines go up shows how the use of that word has grown over time. Words like “behavioural biases”, “behavioural finance”, “investment decision” and “overconfidence” have become more common, especially in recent years. These words began appearing more frequently after 2020, suggesting that an increasing number of researchers are now focusing on these topics.

There are also other words like “decision making”, “financial literacy” and “herding” that show a steady increase. These terms are used when discussing how individuals make financial decisions and how their behaviour can be influenced. Some words are written in different ways, like “behavioural” and “behavioural”, reflecting variations in language usage (American vs. British English), but they mean the same thing.

Figure 6 suggests that the research focus has become more detailed and diverse over time, with increased attention being given to the psychological and behavioural elements of financial decision-making. The steady rise in these terms highlights a growing academic interest and an evolving research landscape in this area.

Figure 7 presents the Conceptual Structure Map generated using the Multiple Correspondence Analysis (MCA) method through Biblioshiny. This visualisation illustrates how keywords from the dataset are conceptually grouped based on their co-occurrence in past studies. Each label represents a keyword, and its position indicates how frequently it appears together in the same study. Keywords that are positioned close to one another are conceptually related and often addressed within the same research context. The shaded triangle outlines a conceptual cluster showing the broader thematic space covered by the dataset.

The connection between financial literacy and behavioural biases is still underdeveloped. Although the term “financial literacy” does appear in the literature, its growth over time has been relatively slow. This suggests that the role of education in shaping or reducing behavioural biases has not been fully explored. There is an opportunity for future research to conduct experimental studies that examine how financial education may influence, reduce or possibly fail to reduce these biases over time.

The map shows several distinct groupings of keywords, such as “investment decisions”, “loss aversion” and “regret aversion”, appear nearby, suggesting a thematic focus on emotional and psychological influences on investment behaviour. Another group includes “financial literacy”, “overconfidence bias”, “decision making” and “structural equation modelling”, which reflects studies that combine knowledge, behaviour and quantitative analysis in decision contexts. Terms like “investments” and “individual investors” appear further apart, indicating studies with a broader or more applied focus on investor profiles and actions. The map captures the underlying intellectual structure of the field, showing that while all terms relate to behavioural finance and decision making, they form multiple interconnected subthemes. This diversity highlights the richness and complexity of current research in this area.

However, while emotional biases and financial knowledge have been well-studied individually, the connection between the two remains largely unexplored. Specifically, how these factors interact to influence actual investor behaviour, especially among individual investors, remains unclear. More research is needed to understand this interaction in real-life settings, which could help make behavioural finance more practical and relatable.

The ‘term co-occurrence network visualisation map’, generated using VOSviewer and based on bibliometric data extracted from Scopus-indexed publications, offers a detailed overview of the key concepts and themes in the literature on behavioural biases and investment decision-making (see Figure: VOSviewer map). This map identifies frequently occurring terms from the titles and abstracts of scholarly articles, visually representing the thematic structure of the field.

Each node in the map represents a term, and its size indicates the frequency with which the term appears in the reviewed literature. For example, the terms “investment decision” (58 occurrences), “study” (58) and “investor” (58) were among the most frequently mentioned, emphasising their foundational role in this research domain. This finding aligns with ^16^ Who highlighted that investment decisions are at the core of studies exploring the impact of behavioural traits. Similarly, ^21^ emphasised the importance of understanding investor behaviour through a behavioural finance lens.

The relevance score complements the occurrence count by indicating the importance of each term in distinguishing between topics. For instance, “Originality value” had a relevance score of 1.67, “Design methodology approach” scored 1.56, and “Practical implication” had a score of 1.47. These high relevance values suggest that these terms are not only standard but also central to differentiating key areas of investigation. According to ^22^ Such methodological aspects are vital in examining overconfidence bias and other psychological tendencies in investment behaviour.

In terms of behavioural themes, terms such as “loss aversion”, “overconfidence”, “representativeness” and “disposition effect” appear frequently, supporting the emphasis placed on cognitive biases across various studies. ^23^ These are closely linked to the practical implications for both investors and policymakers, a connection also noted in recent work by. ^17^

The network structure further groups terms into clusters based on the strength of co-occurrence, illustrating how certain concepts are commonly studied together. For example, one cluster includes “behavioural bias”, “behavioural finance”, and “investment decision”, pointing to core theoretical discussions. Another connects the terms “design methodology approach”, “questionnaire”, and “structured questionnaire”, indicating a methodological focus in empirical studies. Moreover, terms like “practical implication”, “originality value” and “policymaker” highlight the applied aspects and stakeholder relevance emerging in the literature.

Geographically, the presence of “India” (11 occurrences) in the network points to a regional concentration of research, consistent with the findings of, ^17^ who conducted a focused study on Indian stock market investors.

This term, co-occurrence analysis provides a structured and visual way to understand the direction and density of academic work on behavioural biases in investment decisions. By mapping both the frequency and contextual importance of terms, this approach highlights dominant research areas, identifies underexplored topics and supports future research planning. The results are consistent with existing literature examples of ^16, 21^ Moreover, reinforce the importance of linking psychological factors with practical investment behaviour.

While many studies explore the behavioural biases that influence investment decisions, much less work has been done to understand how these biases can be managed or reduced in practice. In particular, there is limited research on the effectiveness of interventions such as financial education, behavioural nudges, or digital tools in real investment settings. Furthermore, a majority of studies focus on specific regions, like India, suggesting a need for broader research across different countries and investor types to capture more diverse insights.

**Figure 9.**
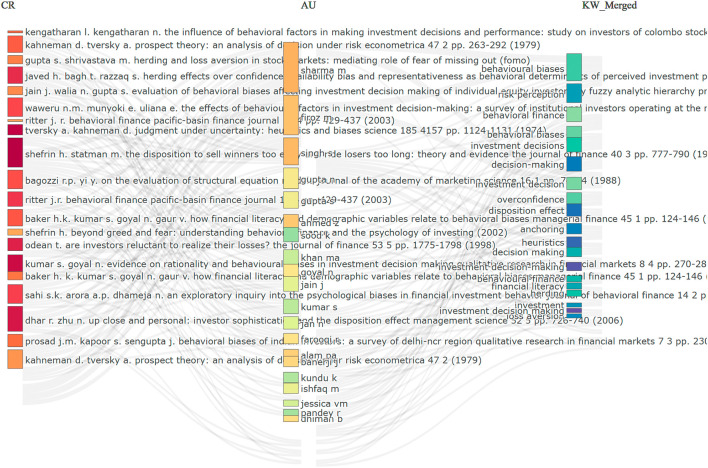
Three field plot. Source: Authors through Biblioshiny.

Figure 9 illustrates the Three-Field Plot, which shows the relationship between cited references (CR), contributing authors (AU), and commonly used keywords (KW). It visually maps how key studies, researchers and research topics are interconnected within the field. On the left side, the plot highlights the most influential cited references. Foundational works such as Kahneman and Tversky’s (1979) ^24^ paper on prospect theory and contributions by Shefrin and Statman appear prominently. These works continue to guide current research in behavioural finance and investment psychology, as evidenced by their frequent citations. In the centre section, the focus shifts to authors who have made multiple contributions to the field. Names like Sharma M., Singh S. and Firoz M. are among the most visible, suggesting they have made several contributions and are central voices in this research area.

On the right side, the plot emphasises key thematic areas through frequent keywords. Terms such as “behavioural biases”, “investment decision”, “behavioural finance”, “overconfidence” and “risk perception” dominate this area. These keywords reflect the core concerns and interests of researchers working in this field.

The connecting lines between these three columns illustrate how specific authors draw upon influential studies and concentrate on certain research themes. This figure provides a clear picture of the intellectual structure of the field, showing which topics are most prominent, who is contributing to them, and which previous studies have laid the groundwork for further research.

**Figure 10.**
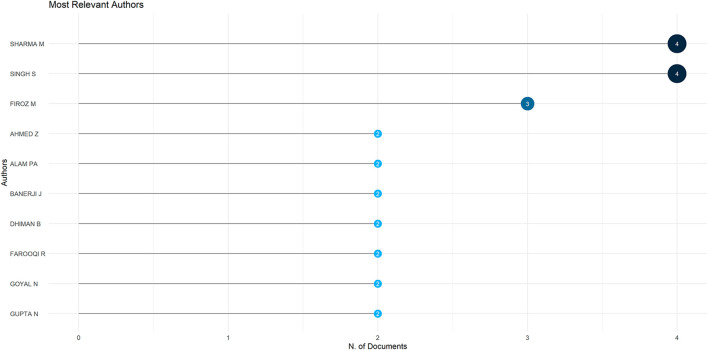
Most relevant authors. Sources: Authors through Biblioshiny.

Figure 10 below displays the researchers who have contributed the highest number of publications within the selected dataset. The frequency of their work suggests they are central figures in shaping the direction and development of this research area.

While the field is grounded in strong foundational studies and widely accepted themes, there is limited exploration of newer topics that reflect today’s changing investment landscape. Areas such as digital investing, social media influence, AI-based financial tools, and generational or cultural differences in investment behaviour are largely underexplored. Additionally, the concentration of work around a few dominant voices and familiar keywords may limit the inclusion of fresh perspectives or emerging issues. Future research could benefit from diversifying its focus to include new technologies, broader demographics and the evolving ways people make investment decisions in the modern world. In other words, scholars could also propose updated models for digital, emerging market, or climate risk settings.

**Figure 11.**
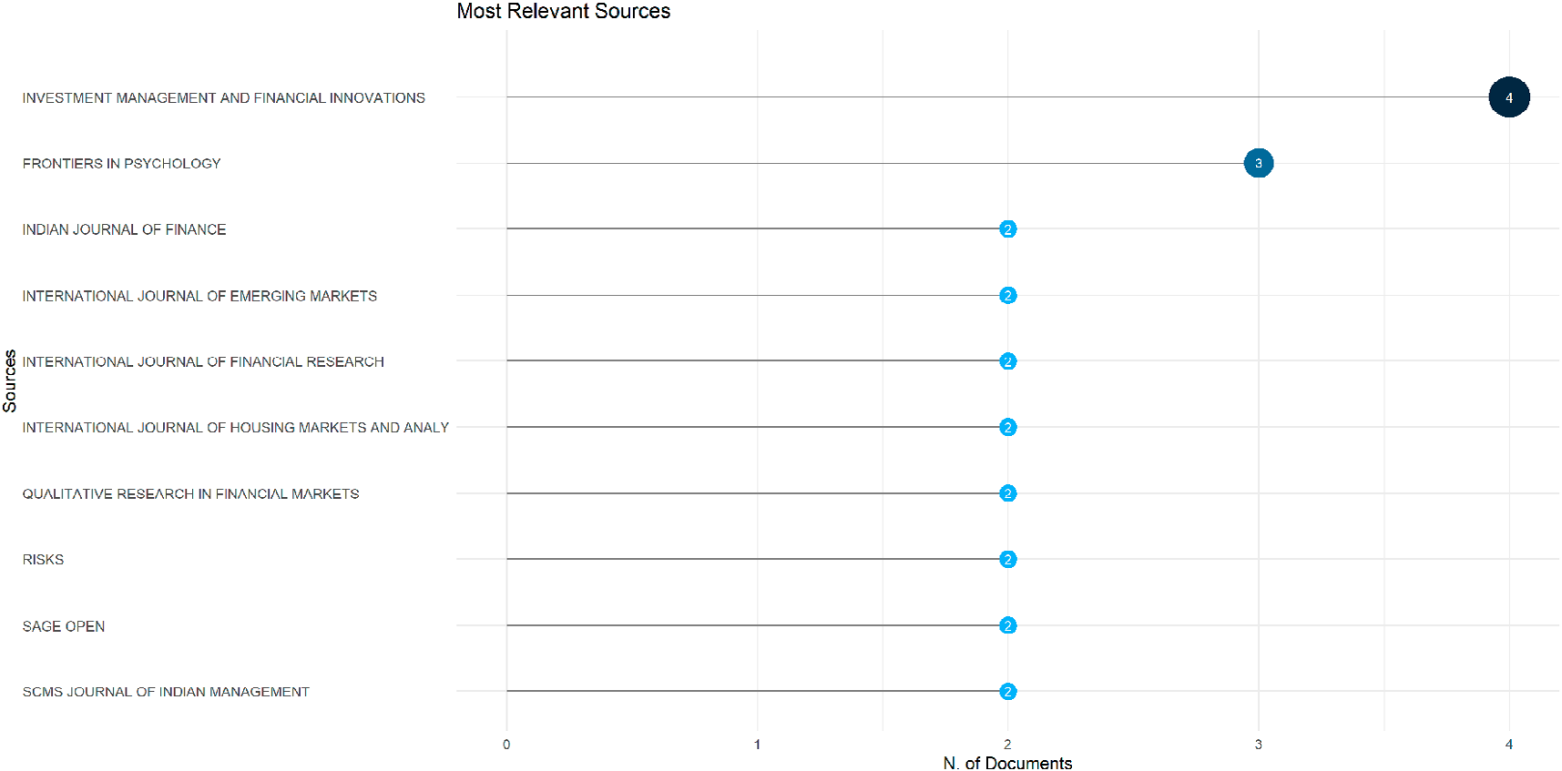
Most relevant sources. Source: Authors through Biblioshiny.

Figure 11 shows which journals have published the most papers in this study. The names of the journals are on the left, and the size of the circle next to each name shows how many papers came from that journal. A larger circle means more papers. The journal “Investment Management and Financial Innovations” has the largest circulation, with four papers, making it the most frequently used journal. “Frontiers in Psychology” follows with three papers. Other journals, such as the “Indian Journal of Finance,” “International Journal of Emerging Markets,” “Risks,” and “SAGE Open,” each have two papers.

These journals belong to various areas, including finance, psychology, and management. This demonstrates that the topic of behavioural finance and investment decision-making is not limited to one field, but is studied from multiple perspectives. According to the Scimago Journal Rank (SJR), some of these journals have strong academic positions. For example, “Frontiers in Psychology” and “Risks” are ranked Q2, indicating they are among the top 50% of journals in their respective fields. “International Journal of Emerging Markets” is also ranked Q2, reflecting its good academic reputation. “Investment Management and Financial Innovations” and “Indian Journal of Finance” are ranked in the Q3 category, which places them in the middle range. “SCMS Journal of Indian Management” holds a Q4 position, indicating it is in the early or developing category of academic influence.

This mix of journals from different quartiles suggests that research on this topic is shared in both well-established journals and newer or growing ones, reflecting a broad interest across the academic community. However, it is worth noting that none of the top journals on this list are ranked in the Q1 category. This means that while the topic is gaining attention, the most frequently used journals for publishing this research are not yet among the highest-ranked in their fields. This could reflect either the emerging nature of the topic or the publishing choices of researchers working in specific regions or disciplines.

However, this also highlights a research gap. Most of the work originates from a small group of authors, and the field has yet to establish a broad or global contributor base. At the same time, the limited presence of Q1-ranked journals indicates that behavioural finance research on investment decisions has not yet fully penetrated the most elite academic spaces. To grow stronger, this area of study needs more diverse authorship and wider publication in high-impact journals. This can help attract global attention, enhance research quality, and bring in new ideas from diverse fields and perspectives.

## Discussion

This review examined how individuals’ behaviour and psychology influence their investment decision-making. It has been found that investors often do not act in an entirely logical or rational manner. Instead, their choices are influenced by different mental shortcuts, also known as behavioural biases. These include being too confident in their knowledge, following what others are doing (herding), and being more afraid of losing money than they are of gaining it. These behaviours are even more common in informal financial settings, where there are fewer rules or professional guidance to help people make informed decisions.

Over time, interest in this topic has increased, particularly over the last decade. Many studies now focus on how these biases affect investors, and most of this research comes from South Asian countries, particularly India. This suggests that researchers are increasingly focusing on the role of psychology in investment, particularly in countries where informal saving and investing are prevalent. However, many areas of the world remain under-researched, and there is a lack of global coverage.

Another critical point is that many studies focus on just a few common biases, like overconfidence and herding. Other significant biases, such as anchoring or regret aversion, are not studied enough. Most research also only explains that biases exist, but it does not try to find ways to solve or reduce them. There is very little research that tests whether interventions such as financial education, reminders (also known as nudges), or digital tools can help people make more informed economic choices. Additionally, many of these studies are published in journals with low impact, so the research is not always reaching a wider audience.

In short, this review demonstrates that we have gained a deeper understanding of how behavioural biases influence investment decisions. However, there is still a lot more to explore. We need to study a broader range of biases, including those from more countries and diverse financial situations, and test real solutions that can help investors. By doing this, future research can help people make more informed financial decisions, especially in areas where formal financial support is limited.

### Future research agenda

Based on the findings of this review, several key points can be identified for future research, practice, and policy.

1. Diversifying the biased portfolio

First, researchers should expand their focus beyond the most commonly studied biases. While overconfidence, herding, and loss aversion are essential, other biases, such as anchoring, mental accounting, regret aversion, and emotional reasoning, also significantly influence investment behaviour. Studying a wider range of biases will provide a more comprehensive and realistic picture of how investors think and act, particularly in unpredictable or high-risk environments.

2. Contextual & cultural expansion

Second, future research should include a more diverse set of participants and financial settings. Most current studies are limited to certain countries and often focus on formal markets. However, many people around the world make financial decisions in informal or semi-formal settings such as rotating savings groups, community lending schemes or digital investment platforms. These environments often lack proper regulation and financial education, which can exacerbate biases. Including such contexts in future studies can help design more effective interventions for individuals who are most vulnerable to financial mistakes.

3. Debiasing interventions and methodological innovation

Third, there is a strong need for studies that go beyond describing problems and start testing solutions. Researchers should investigate whether interventions, such as financial literacy programs, nudges (including reminders or prompts), or user-friendly decision tools, can mitigate the impact of harmful biases. Experiments, pilot programs, and digital platform trials can help test what works in real-life settings. These findings can then be shared with educators, app developers and financial advisors who work directly with investors. Additionally, using various research methods, such as combining surveys with experiments or analysing digital behaviour data, can help researchers track changes over time and identify sustainable interventions.

4. Publication quality & collaboration

Another recommendation is to strive for higher-quality research and broader impact. Publishing in top-ranked journals and presenting findings in interdisciplinary forums will help the research reach a broader audience and be taken seriously by both academic and policy communities. Collaborating with psychologists, technology experts, and financial professionals can lead to richer insights and better solutions. In addition, translating academic findings into practical guidelines or policy briefs can help ensure that the knowledge is used outside of universities and contributes to real-world improvements.

5. Integrating risk perception, financial literacy, and support tools

Ultimately, for policymakers and practitioners, the findings of this review suggest that more effort should be devoted to educating investors in relatable and straightforward ways. Programs should explain not only how financial tools work but also how the mind can trick us into making poor decisions. Visual aids, short videos and mobile-based content could make such education more accessible. Financial service providers can also design platforms that gently alert users when they may be making impulsive choices or falling into common traps. Governments and community organisations should collaborate to enhance transparency in informal financial systems, enabling individuals to access accurate information before making informed investment decisions.

6. Towards a more inclusive and informed investment future

In conclusion, understanding and addressing behavioural biases in investment decisions is not just a theoretical task but a practical necessity. By enhancing the scope and quality of research and making its findings more valuable and accessible, we can support more informed financial decisions, particularly for individuals who rely on informal and unregulated financial systems. This work is key to building a more inclusive, informed and resilient investment environment for the future.

## Data Availability

The main data for this article are the bibliographic references, which are already included in the References section. In addition to that, the extended data for this study, including the PRISMA checklist, flow diagram and other supplementary materials, are openly available in Zenodo at; https://doi.org/10.5281/zenodo.17284122 ^25^ Data are available under the terms of the Creative Commons Attribution 4.0 International All data underlying the results are already part of this article, and no additional source data were required. The software used for analysis is available from: https://posit.co/download/rstudio-desktop/, https://www.vosviewer.com/, https://www.zotero.org/
