# Supplementary material for: Unpacking Investor Psychology: A Systematic Review and Meta-Analysis of Behavioural Biases Shaping Investment Decisions*
Source: F1000Res. 2025 Oct 21;14:1146. [Version 1] doi: 10.12688/f1000research.168166.1 (PMC12576316; doi:10.12688/f1000research.168166.1)
Supplement: Peer review history

# Reviewer report: Reviewer response for version 1

**DOI:** 10.5256/f1000research.185331.r426000

**Published:** 2025-12-11

**Authors:** Sujata Kapoor

**Affiliations:** 1 Jaypee Business School, Noida, Uttar Pradesh, India

A total of 63 empirical papers were selected for the systematic literature review (SLR) using the PRISMA technique, ensuring a transparent screening process. The paper is well-structured and visually enriched, presenting a clear tree map of keywords, word frequency trends over time, and a detailed conceptual structure map, which together provide a comprehensive overview of the thematic evolution within the field.

 Moreover, the study highlights country-wise list of research papers, reporting the specific percentages and citation-based valuations attributed to each country.

 Henceforth, illustrating global research contributions and influence. Amongst the major behavioural biases identified across the reviewed literature, overconfidence and herding emerged as the most dominant themes.

 The current paper covers the literature review of herding and overconfidence biases with respect to individual investors' decision making, the same study can also be extended to cover these  biases with respect to the financial professionals.Herding and overconfidence has been found to be dominant biases in the literature with respect to financial professionals.

Are the rationale for, and objectives of, the Systematic Review clearly stated?

Yes

Is the statistical analysis and its interpretation appropriate?

Yes

If this is a Living Systematic Review, is the ‘living’ method appropriate and is the search schedule clearly defined and justified? (‘Living Systematic Review’ or a variation of this term should be included in the title.)

Partly

Are sufficient details of the methods and analysis provided to allow replication by others?

Yes

Are the conclusions drawn adequately supported by the results presented in the review?

Yes

Reviewer Expertise:

Corporate Finance, Behavioral Finance and Neurofinance

I confirm that I have read this submission and believe that I have an appropriate level of expertise to confirm that it is of an acceptable scientific standard.

# Reviewer report: Reviewer response for version 1

**DOI:** 10.5256/f1000research.185331.r425994

**Published:** 2025-10-30

**Authors:** Nirma Sadamali Jayawardena

**Affiliations:** 1 University of Bradford, Bradford, UK

The article selection process in this study adhered to the four steps outlined in the PRISMA flow diagram: identification, screening, eligibility and inclusion. The concepts employed to search were “Behavioural Biases” and “Investment Decisions”, which were strategically chosen to capture relevant studies in the field. This is a very good article and would recommend the acceptance as it is.

Are the rationale for, and objectives of, the Systematic Review clearly stated?

Yes

Is the statistical analysis and its interpretation appropriate?

Yes

If this is a Living Systematic Review, is the ‘living’ method appropriate and is the search schedule clearly defined and justified? (‘Living Systematic Review’ or a variation of this term should be included in the title.)

Yes

Are sufficient details of the methods and analysis provided to allow replication by others?

Yes

Are the conclusions drawn adequately supported by the results presented in the review?

Yes

Reviewer Expertise:

consumer behaviour marketing and advertising

I confirm that I have read this submission and believe that I have an appropriate level of expertise to confirm that it is of an acceptable scientific standard.

# Reviewer report: Reviewer response for version 1

**DOI:** 10.5256/f1000research.185331.r425997

**Published:** 2025-10-30

**Authors:** Isuru Koswatte

**Affiliations:** 1 University of the West of Scotland, Paisley, Scotland, UK

This systematic review on investor psychology and behavioural biases shaping investment decisions is an exceptionally well-executed and thoughtfully organised study that reflects a deep understanding of the field. The authors clearly articulate a strong rationale for undertaking the review, effectively identifying key research gaps in the literature on behavioural finance and highlighting the need for a systematic synthesis of existing evidence. The objectives are well-defined and directly aligned with the study’s purpose, providing a clear and logical flow throughout the paper. The methodological approach is sound and transparent, with well-documented procedures for database selection, search strategy, screening, and inclusion criteria, ensuring both rigour and reproducibility. The integration of bibliometric and meta-analytic techniques is a major strength, adding depth to the analysis and offering fresh insights into how behavioural biases—such as overconfidence, herding, and loss aversion—have been studied across different contexts and economies. The presentation of findings is clear and engaging, supported by well-designed visual analyses that help the reader grasp complex relationships and thematic trends in the literature. The authors’ discussion is particularly strong, as it links the review’s findings to broader debates in behavioural finance, investor behaviour, and emerging market dynamics. The conclusions are well-supported by the results and thoughtfully consider both theoretical and practical implications, offering valuable guidance for future research and policy. Overall, this review represents a significant and timely contribution to understanding how psychological factors shape investment decisions, showcasing methodological integrity, analytical depth, and clear scholarly impact.

Are the rationale for, and objectives of, the Systematic Review clearly stated?

Yes

Is the statistical analysis and its interpretation appropriate?

Yes

If this is a Living Systematic Review, is the ‘living’ method appropriate and is the search schedule clearly defined and justified? (‘Living Systematic Review’ or a variation of this term should be included in the title.)

Yes

Are sufficient details of the methods and analysis provided to allow replication by others?

Yes

Are the conclusions drawn adequately supported by the results presented in the review?

Yes

Reviewer Expertise:

My expertise are in Leadership, entrepreneurial resilience and also SME development

I confirm that I have read this submission and believe that I have an appropriate level of expertise to confirm that it is of an acceptable scientific standard.

# Reviewer report: Reviewer response for version 1

**DOI:** 10.5256/f1000research.185331.r426001

**Published:** 2025-10-28

**Authors:** S.N.R. Fiona Dias

**Affiliations:** 1 Department of Banking and Finance, Faculty of Business Studies and Finance, Wayamba University of Sri Lanka, Kuliyapitiya, Sri Lanka

Overall Summary of the Article

 This article systematically reviews existing research on how behavioural biases shape investment decisions across formal and informal financial settings. Using the PRISMA framework and Scopus database, 63 empirical studies (1999–2025) were analysed with bibliometric tools such as VOSviewer and RStudio. The findings reveal growing research attention, particularly in South Asia, with overconfidence, herding, and loss aversion emerging as dominant themes. Less-studied biases like anchoring and regret aversion highlight significant research gaps. The review concludes by proposing a future research agenda focused on broadening contextual coverage, testing debiasing interventions, and enhancing methodological and interdisciplinary quality.

 Rationale and Objectives;

 The rationale for the review is well grounded in behavioural finance theory, and the objectives are stated clearly through relevant research questions.

 Methodological Transparency;

 The methodology is rigorous, transparent, and largely replicable. The detailed explanation of the search strategy, inclusion/exclusion process, and use of analytical tools demonstrates a strong understanding of systematic review protocols. 

 Statistical analysis and its interpretation;

 The statistical and bibliometric analyses are appropriate, clearly explained, and effectively visualized. The results are logically interpreted and align well with the study’s objectives.

 Conclusions

 The conclusions were clearly derived from the patterns, trends, and bibliometric evidence identified in the review and are presented in the discussion. The discussion section appropriately balances summarizing what is known, identifying what is missing, and recommending logical next steps.

Are the rationale for, and objectives of, the Systematic Review clearly stated?

Yes

Is the statistical analysis and its interpretation appropriate?

Yes

If this is a Living Systematic Review, is the ‘living’ method appropriate and is the search schedule clearly defined and justified? (‘Living Systematic Review’ or a variation of this term should be included in the title.)

No

Are sufficient details of the methods and analysis provided to allow replication by others?

Yes

Are the conclusions drawn adequately supported by the results presented in the review?

Yes

Reviewer Expertise:

Finance, Financial Inclusion, Digital Finance, Sustainable Finance, Investment Behaviour

I confirm that I have read this submission and believe that I have an appropriate level of expertise to confirm that it is of an acceptable scientific standard.
